# Hydroxyethyl starch 130/0.4 and sodium chloride injection as adjunctive therapy in patients with cerebral hypoperfusion

**DOI:** 10.1186/1471-2377-12-127

**Published:** 2012-10-30

**Authors:** Junliang Han, Fang Yang, Wenrui Jiang, Guangyun Zhang, Zhirong Liu, Xuedong Liu, Feng Xia, Ya Bai, Junhong He, Manxiang Chao, Gang Zhao

**Affiliations:** 1 Department of Neurology, Xijing Hospital, No. 15 West Changle Road, Xi’an, China, 710032; 2 Department of Cardiology, Tangdu Hospital, No. 1 Xinsi Road, Xi’an, China, 710038

## Abstract

**Background:**

Both **s**evere stenosis and completed occlusion in internal carotid artery or its distal branches have been considered the main reasons of cerebral hypoperfusion, which contributes to the washout disturbances of embolism in low perfusion territories distal to stenosis. An aggravated hypoperfusion state in certain brain region may induce ischemic stroke and further cognitive decline. However, the effective medication for cerebral hypoperfusion is largely unsettled.

**Methods/design:**

By using computed tomography perfusion (CTP) imaging, the trial will evaluate the effectiveness, safety and tolerability of hydroxyethyl starch (HES) 130/0.4 for patients with extra-/intra-cranial artery stenosis and cerebral hypoperfusion. From 5 neurological inpatient wards, 300 patients will be randomly recruited for administered routine medications plus intravascular volume therapies using the equal volume of HES 130/0.4 or 0.9% sodium chloride solution. Cerebral hypoperfusion state after 7-day intervention is the primary outcome measure. The secondary outcome measures includes, impaired renal function, abnormal heart function, hematological changes, neurological dysfunctions and cerebrovascular events in peri-intervention period and/or 3-month follow-up. The sample size will allow the detection of a two-sided 5% significance level between groups in the endpoint with a power of 80%.

**Discussion:**

The trial would provide important efficacy and safety data on the intravascular administration of HES 130/0.4 in patients with unilateral cerebral hypoperfusion. The effects on kidney function, heart function, coagulation, neurological function and cerebralvascular events will be assessed.

**Trial registration:**

ClinicalTrials.gov (Identifier: NCT01192581)

## Background

### Large-artery stenosis-induced cerebral hypoperfusion

As a subtype of the TOAST or CISS classification for ischemic stroke [1,2], large-artery atherosclerosis commonly induces internal carotid artery (ICA) or its distal branches (middle cerebral artery, MCA) stenosis. Severe artery stenosis is the major prognostic stroke risk factor in patients with symptomatic and asymptomatic ICA/MCA disease, and would place hemodynamic stress on the brain circulation. Among asymptomatic patients, increasing frequencies of transient ischemic attack (TIA) and stroke is related to grades of arterial stenosis. In patients with symptoms including transient monocular blindness, TIA and minor strokes, stroke risk highly related with the severity of ICA/MCA stenosis [3,4].

Cerebral hypoperfusion is deemed as an important origin contributed to ischemic stroke or reversible neurological dysfunctions in patients with extra-/intra-cranial arterial stenotic or occlusive disease [5]. Large-artery-stenosis induced cerebral hypoperfusion leads to blockage in smaller distal artery, which would decrease perfusion in the local region. In some cases, embolic materials from proximal artery might appear washout disturbances to increase the degree of artery stenosis. A prolonged failure to deliver oxygen and sugar-rich blood to brain tissue causes a metabolic impairment of neural cells, in severe conditions, irreversible cells death, i.e. cerebral infarction.

### Management of cerebral hypoperfusion

Treatments of ICA/MCA stenosis and secondary stroke-prevention related to intracranial arteries atherosclerosis have been extensively explored under numbers of investigations [6]. However, enough importance has not been attached to the management of cerebral hypoperfusion yet. It is well known that tPA is almost the only effective treatment for patients with acute ischemic stroke, which improves the perfusion state of ischemic penumbra by dissolving the blood clot. Because of the narrow therapeutic window, the use of tPA is largely restricted. Apart from this, antiplatelet therapy, statins therapy and risk factor modifications, as routine medications, are recommended for patients with large-artery atherosclerosis. In invasive treatments, carotid endarterectomy (CEA) is recommended for symptomatic patients with moderate to high grade stenosis (50%–99%). Carotid angioplasty and stenting (CAS) has been indicated as a therapeutic alternative to CEA [7,8], which exerts improving perfusion state in cerebral hypoperfusion region [3,9]. However, the role of CAS remains uncertain and controversial for the asymptomatic patients with ICA stenosis. Recently, researchers reported an anti-migraine agent, lomerizine, could contribute to restoration of cerebral hypoperfusion by SPECT [10].

Theoretically, volume therapy attempts to improve cerebral perfusion of ischemic regions through optimization of hemorheological parameters and increment in cardiac output and blood pressure [11,12]. A previous study [13] confirmed the safety of hypervolemic therapy; however, the sample size was too small to show the clinical efficacy statistically. Furthermore, unfavorable results of volume therapy in acute ischemic stroke was reported by a meta-analysis [14] on survival or neurological function evaluation in 18 clinical trials. During recent decades, colloidal solutions are extensively used in the volume therapy of ischemic stroke in many countries [15]. However, the effect of medication on non-acute cerebral hypoperfusion state is still largely unsettled.

### The clinical effects of hydroxyethyl starches

Hydroxyethyl Starches (HES) is one of the worldwide-used colloidal solutions for decreasing hematocrit and whole-blood viscosity without significant effects on plasma viscosity and red-cell aggregation [16]. HES is classified according to the molecular weight (MW) into high MW (450±480 kDa), medium MW (130–200 kDa), and low MW (40–70 kDa) starch preparations, which exert different therapeutic and adverse effects respectively.

In comparison with other starch preparations, HES 130/0.4 marked for the indication ‘intravascular volume expansion and hemodilution’ in worldwide (Summary of Product Characteristics for Voluven, Fresenius Kabi) and showed advantages in the brain, such as an improvement of hemorheologic parameters, an earlier increase of tissue oxygen tension and a safety in large-dose infusion [17,18]. Mammalian study [19] further indicated that HES 130/0.4 played an anti-inflammatory role in non-brain tissues. However, the effect of HES 130/0.4 on cerebral hypoperfusion state is still unclear.

### Objectives of the study

The objectives of this study are to evaluate the effectiveness, safety and tolerability of consistent dosages of HES 130/0.4 administrated intravenously for 7 days in patients with cerebral hypoperfusion in unilateral ICA or MCA territory. We expect that the results will indicate the effect of HES 130/0.4 on cerebral hypoperfusion.

## Methods/design

### Participants

Total 300 inpatients will be recruited through 5 neurology wards of general hospitals in Xi’an, China. Three subtypes of patients will be enrolled: I, non-acute ischemic stroke (time of stroke onset: 72 hours to 3 weeks); II, TIA with cerebral hypoperfusion; III, asymptomatic cerebral hypoperfusion. Outpatients showed abnormal results in carotid color Doppler and transcranial Doppler (temporal bone window) in routine physical examination, would be enrolled as asymptomatic candidates for further evaluation of cerebral perfusion state.

Prior to data collection, ethics approval have been achieved through the Medical Ethical Reviewing Committee of the Fourth Military Medical University Medical Center. Informed consent will be supported by a patient (or next to kin) information leaflet in Chinese. A consulted meeting will be developed by a study physician to ensure patients and their families understand the study procedure and consent to participation in the trial [20].

### Study design

Multicenter, randomized, paralleled, double-blinded trial with concealed allocation of patients with cerebral hypoperfusion 1:1 to volume therapy using 6% HES 130/0.4 in 0.9% sodium chloride (Voluven, Fresenius Kabi, Germany) or 0.9% sodium chloride solution (normal saline, NS), is stratified by presence of stroke or not. We use digital subtraction angiography (DSA) to verify the severity of ICA or MCA stenosis [21], and evaluate cerebral hypoperfusion in unilateral intracranial artery territories by time to peak (TTP), one of parameters in CTP imaging [22].

### Sample size estimates

The results from our preliminary study were used to calculate the sample size (unpublished data). The mean difference and standard deviation of TTP after HES 130/0.4 were 19.83 and 10.21, respectively. A two-sided 5% significance level and 80% power were considered, and the following equation was used:

$$n=\frac{2\times {\left(\zeta_{\alpha /2}+\zeta_{\beta }\right)}^{2}\times \sigma^{2}}{\delta^{2}}$$

Approximately 60 participants in each group were calculated to be required. A 20% attrition rate is anticipated over the 2-year study period, resulting in about 300 participants in total four groups as a final estimated sample size.

### Randomization procedures

Random number will be generated by a computerized random-number generator at statistics research office, Fourth Military Medical University. A clinical research associate will make opaque blinded envelopes (with consecutive numbers) and deliver them to each participating center. Random allocation will be performed within 12 hour after a patient enrolled. The block size and treatment-assignment table will not be available to the researchers until the end of the trial.

### Inclusion criteria

All patients presenting to the study center are eligible for inclusion in the study if they fulfill the following requirements:

1. Age: 18 to 75 years

2. The degree of ICA/MCA stenosis is greater or equal to 50% confirmed by DSA

3. Before HES treatment, CTP imaging shows that TTP in MCA territory is extended (≥15%) in comparison with that in corresponding area of contralateral hemisphere

4. If cerebral infarction appears,

① Time of stroke onset: 72 hours-3 weeks

② Magnetic resonance imaging (MRI) + diffusion weighted imaging (DWI) shows ischemic stroke due to unilateral ICA or MCA stenosis infarcts

③ NIH stroke scale: 3–20

5. Written consent obtained from patient or the proxy

### Exclusion criteria

Patients are excluded from the study if they met any of the following criteria:

1. Allergy to the components of HES

2. Clinical features including severe dehydration, overhydration and hydrocephalus

3. Chronic liver disease (ALT > 120 or AST > 120)

4. Chronic renal disease (Scr > 150 μmol/L)

5. Severe heart failure which correspond to NYHA heart failure classification class III or IV, or serious arrhythmia, myocardial infarction

6. Intracranial hemorrhage or structural brain lesions which can mimic stroke (e.g. cerebral tumor) by CT or MRI brain scanning.

7. Pregnant and lactating women

8. Patients suffered from epilepsy or mental sickness

9. Alcoholism or drug abuse

10. HES or other artificial colloidal solution was used within 3 months.

11. Patients participate in other clinical trial within 6 months

12. Contraindication to CT perfusion imaging (i.e. contrast allergy, metformin use or Creatinine >160 μmol/l)

13. Thrombus in lower limb vein

### Primary outcome measure

The alternation of TTP in CTP image defined as cerebral hypoperfusion-improvement 7 days after volume therapy will be the primary outcome measure.

### Secondary outcome measures

1. Cardiac-/cerebral-vascular events in three-months

2. Impaired kidney function after randomization

3. Impaired heart function at 3-day and 7-day after randomization

4. Blood-coagulation changes at 7-day after randomization

5. Neurological function scores (Table 1) at 7-day and 3-month after randomization

**Table 1:** Outcome variables and assesment schedule

**Outcome variables**		**Baseline**	**Intervention period**		**Post**-**intervention**	**3 month follow**-**up**
			**Each day**	**5**^**th**^**day**		
***Primary outcome measure***						
TTP		✓			✓	
***Secondary outcome measures***						
Symptoms (Neuro-check)		✓	✓		✓	✓
Blood pressure		✓	✓		✓	✓
ECG		✓	✓		✓	
DEC		✓			✓	
Blood/plasma viscosity		✓		✓		✓
Blood coagulation profile		✓		✓	✓	✓
CCr		✓		✓	✓	
Lipid profile		✓			✓	✓
MMSE		✓			✓	✓
**Patients With Stroke**	NIHSS	✓			✓	✓
	BI	✓			✓	✓
	mRS	✓			✓	✓

*ECG*, Electrocardiogram; *DEC*, Doppler Echocardiography; *CCr*, Creatinine clearance; *MMSE*, Mini-mental State Examination; *NIHSS*, National Institutes of health Stroke Scale, *BI*, Barthel Index; *mRS*, modified Rakin Scale.

### Study procedures

**Figure 1:**
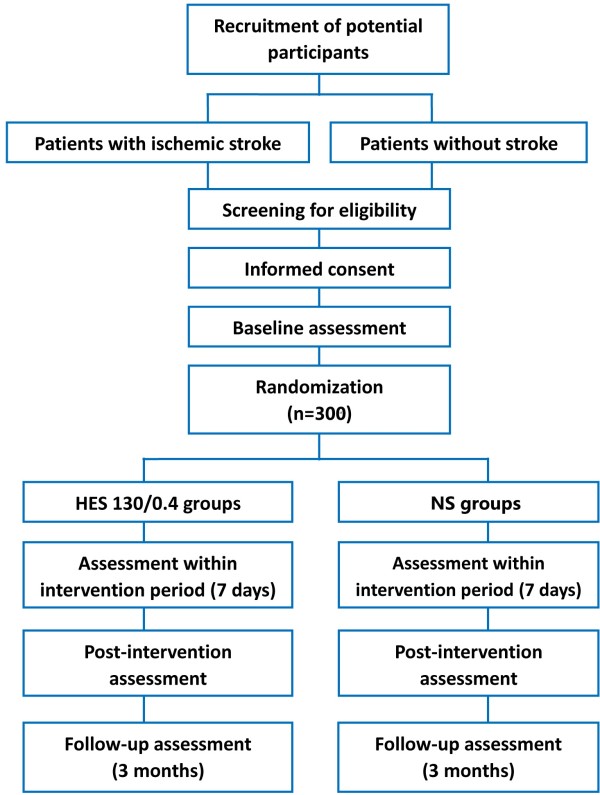
Flowchart outlining the trial protocol.

Figure 1 shows the trial procedure. Participants with suspected unilateral ICA or MCA stenosis will firstly receive CT and CTP brain scanning to exclude the intracranial hemorrhage and be confirmed their cerebral perfusion state. Patients with unilateral cerebral hypoperfusion will be further evaluated by physicians in participating hospitals according to a series of evaluation procedure, including history taking, complete physical examination, radiology tests and relative laboratory tests (Table 1). For radiology tests, MRI brain scanning will be applied to confirm whether a consistent infarct exist in hypoperfusion region or not, and DSA will be performed to determine the degree of artery stenosis/occlusion. Once a standardized, structured interview is performed, the data will be recorded in the case report form for each patient. All clinical data, biological samples and radiological images would be sent to the central study site where a cerebrovascular neurologist would review the data. For the patients with non-acute ischemic stroke, National Institutes of Health Stroke Scale, Barthel Index and modified Rakin Scale will be performed by an individual researcher. Demographic, medical, social, and behavioral variables will be determined along with baseline medications. Anthropometry will be conducted using standardized equipment calibrated on a daily basis.

Participants will be randomized to one of two interventions: HES 130/0.4 and NS with two daily dosages in each intervention, i.e. 500 ml and 1000 ml, for 7 consecutive intervention days. A range of outcome measures (Table 1) will be used to ensure evaluation of relevant variables, based on the primary and secondary aims of the trial, clinical relevance and feasibility. Patients will be enrolled initially in the 500 ml groups. If more than 30% patients experience intolerable adverse events, the trial should be stopped. Following successful completion of 500 ml dose study, patients should be enrolled in the 1000 ml groups.

According to our preliminary study, 60% (15/25) of patients were found infarct responsible focuses in MCA territory by MRI brain scanning. To ensure comparable groups in terms of neurological status, group assignment will be stratified by having infarct responsible focuses or not. An individual not associated with the trial will use a computerized random number generator to randomly assign subjects to group. Allocation concealment will be ensured by using opaque, a sealed envelope containing group assignment.

### Intervention

All participants will receive routine medications including antiplatelet therapy, statins therapy, risk factor modification and others if necessary. Intervention group will receive a prescribed dose of 0.6% HES 130/0.4 in 7 intervention days. Control group will be treated the same volume of NS each day in the intervention period. The initial 10–20 ml will be infused slowly, keeping the patient under close observation.

Although, the infusion dose of HES 130/0.4 up to 33 mL/kg/day is most commonly used in clinical trials and repetitive large doses of up to 70 ml/kg/day have been reported in the literatures [23-26]. Considering potential risks of overloading the circulatory system and increasing brain edema by overdose and the median cumulative HES dose (2465 ml, range 328 ml to 6229 ml) in resuscitation [27], two dose groups (500 ml/day and 1000 ml/day) have been chosen as cumulative dose of 3500 ml and 7000 ml.

### Statistical analysis

Data will be exported into SPSS V15.0 software for subsequent analyses. A statistical analysis plan will be drafted at the start of the project and all analyses will be carried out after masking allocation. Independent samples t-tests, Mann–Whitney U tests and Chi-square test of association will be used as appropriate to compare groups at baseline. ANOVA or logistic regression will be used to determine significance of the results obtained.

## Discussion

Because of the lack of reliable clinical data, the effect of volume therapy (HES 130/0.4) on cerebral hypoperfusion due to severe ICA/MCA stenosis or occlusion is largely unsettled. In this trial, three types of patients with cerebral hypoperfusion will be recruited: non-acute ischemic stroke, TIA and asymptomatic patients. Although volume therapy could possibly decrease blood viscosity and may increase cerebral perfusion or oxygen delivery, previous studies [13,24,28] focused on acute ischemic stroke with treatment windows from 6 hours to 48 hours resulted no improvement in outcome or survival at the endpoints. In this trial, we’d like to enroll patients with non-acute ischemic stroke (time of stroke onset: 72 hours to 3 weeks) for the following reasons: 1. variable cerebral hypoperfusion state and higher risk of hemorrhagic transformation may appear in patients with acute ischemic stroke (< 72hours); 2. from 72 hours to 3 weeks of stroke onset, patients’ emotional state and cerebral auto-regulation are relatively stable. Cerebral hypoperfusion with severe intracranial atherosclerosis is one of common causes of ischemic stroke and TIA. Unless the collateral blood supply is sufficient to prevent ischemia, multiple remote spot-like infarctions may occur within the hypoperfused brain territory. Certainly, the superiority of surgical interventions over medical therapy has been confirmed in symptomatic patients with severe ICA stenosis [8,29]. However, for the asymptomatic patients with severe or moderate ICA stenosis, the role of surgical interventions remains uncertain. For the patients with ICA or MCA occlusion, medication could be the only practical therapy. Unfortunately, few effective medical treatments have been developed for the treatment of cerebral hypoperfusion. In this trial, we will use NS as control to compare the effect of HES 130/0.4 on the improvement of cerebral hypoprefusion in MCA territory. By using CTP imaging to confirm the brain perfusion state [30], this trial will contribute to study the effectiveness, safety and tolerability of safety of HES 130/0.4 for patients with chronic cerebral hypoperfusion.

## Competing interests

The authors declare that they have no competing interests.

## Authors’ contributions

ZG: the principal investigator for this project who led the conceptualization, design, funding applications and made a strategic decision of this research protocol. HJ and YF: co-led the conceptualization, design, development, and implementation of this research protocol and contributed to the writing of this manuscript. XF, HJ, CM: contributed to the design, development, and implementation of this research protocol. JW, LX: led the development of the data management protocol and statistical analysis plan. BY: assisted in the design of this protocol. All authors read and approved of the manuscript.

## Pre-publication history

The pre-publication history for this paper can be accessed here:

http://www.biomedcentral.com/1471-2377/12/127/prepub
